# Prospective Follow-up of Intramedullary Slitlike Cavities: A Consecutive Series of 48 Patients

**DOI:** 10.3389/fneur.2020.00495

**Published:** 2020-06-12

**Authors:** Matthieu Faillot, Silvia Morar, Sebastien Delphine, Mounir El-Mendili, Denis Ducreux, Fabrice Parker, Nozar Aghakhani

**Affiliations:** ^1^Neurosurgery Department, Bicêtre University Hospital, Assistance Publique Hôpitaux de Paris (APHP), Université Paris-Sud, Le Kremlin-Bicêtre, France; ^2^CRMR C-MAVEM (Centre de reference Maladies Rares Chiari-Syringomyélie et Malformations vertébrales et médullaires rares/Reference Center for Rare Diseases: Chiari-Syringomyelia and Rare Malformations of the Spine and Spinal Cord), Paris, France; ^3^Biomedical imaging laboratory (LIB, Laboratoire d'Imagerie Biomédicale), CNRS, UMR 7371, INSERM, UMR-S 1146, Sorbonne Université, Paris, France; ^4^Neurology Department, Icahn School of Medicine at Mount Sinaï, NewYork, NY, United States; ^5^Neuroradiology Department, Bicêtre University Hospital, Assistance Publique Hôpitaux de Paris (APHP), Université Paris-Sud, Le Kremlin-Bicêtre, France

**Keywords:** MRI, intramedullary slitlike cavity, spinal cord, syringomyelia, DTI

## Abstract

**Object:** Predicting whether intramedullary slitlike cavity (SC) will worsen over time or remain stable is an outstanding clinical challenge. The aim of this study was to identify early features of SC (clinical and magnetic resonance imaging [MRI] findings).

**Methods:** We prospectively included all patients referred to our institution following the discovery of a SC and divided them in two groups: typical SC (defined as a cavity spanning fewer than three vertebrae, not enlarging the spinal cord, and located at the midline between the anterior third and posterior two-thirds of the spinal cord) or atypical SC (all others). Clinical evolution and changes in MRI features were evaluated during follow-up. In some patients, diffusion tensor imaging was performed and cervical cord cross-sectional area was analyzed.

**Results:** A total of 48 consecutive patients were included in the study. The mean follow-up was 58 months. Of the seven patients presenting with deficits at first consultation, two worsened and five remained stable. Of the 41 patients without deficits, seven worsened and 34 remained stable. None of the patients developed severe motor deficits or experienced enlargement of the cavity; 7% of patients who presented with typical SC worsened compared with 35% with atypical SC. The negative predictive value was 0.93 (*P* = 0.02).

**Conclusion:** Most patients remained stable and a subset of patients developed minor motor deficits. For clinical management, we propose surveillance of patients with a typical SC and close follow-up of those with an atypical SC and/or presenting with deficits.

## Introduction

### Background/Rationale

Neurologists and neurosurgeons regularly encounter patients referred following the discovery of an intramedullary slitlike cavity (SC). These patients usually undergo magnetic resonance imaging (MRI) for neck or upper limb pain mimicking cervical radiculopathy; an abnormal image may also be discovered as an incidental finding. These cavities are sometimes referred to as hydromyelia (1, 2) or as a dilation of the central canal (3).

While physical examination frequently reveals minor abnormalities, it is important to determine whether patients will remain stable or will experience neurological deterioration. There is currently a lack of consensus regarding the modalities used in follow-up and the information delivered to patients.

### Objectives

The aim of this study was to prospectively analyze the natural history and imaging features of SCs. We hypothesized that patients at risk of neurological deterioration who should be closely followed up are identifiable by spinal cord MRI.

## Materials and Methods

### Study Design and Setting

This is a prospective study performed at one academic neurosurgical center which is a reference center for syringomyelia and related diseases. Patients were included between January 2015 and January 2017.

### Participants and Study Size

We included all consecutive patients referred for a SC. All patients with evidence of Chiari malformation, demyelinating disease, tumor related syringomyelia and post traumatic syringomyelia were excluded. The study was approved by the Ethics Committee of the Pitié-Salpêtrière Hospital (ID RCB: 2013-A01547-38, Paris, France). We obtained written informed consent for all participants.

### Variables

American Spinal Injury Association (ASIA) score was determined and pain was evaluated using a numeric pain rating scale (NPRS) ranging from 0 (no pain) to 10 (worst pain possible). Motor function and the presence of urinary symptoms were assessed at baseline and during follow-up by a neurosurgeon MF,SM,NA).

**Figure 1:**
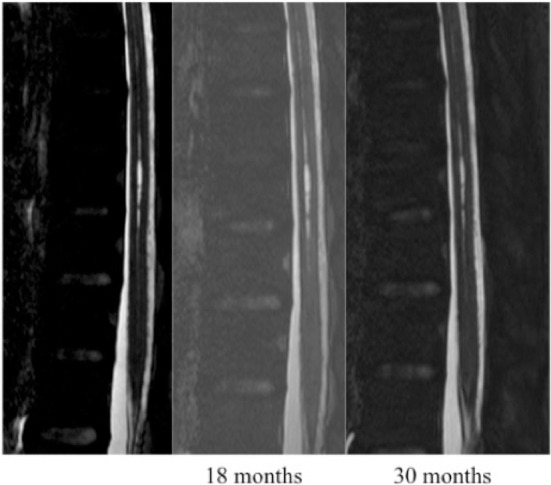
Patient with a typical slitlike cavity.

**Figure 2:**
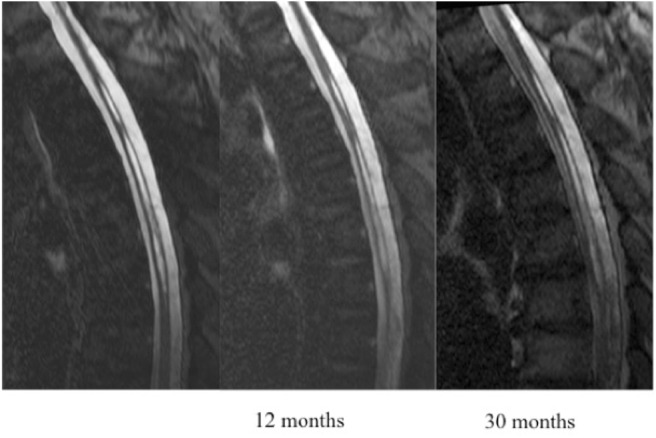
Patient with an atypical slitlike cavity.

Based on MRI T2/DRIVE analysis we described two type of cavities: A typical SC was defined as one meeting all of the following criteria: located at the midline (in the axial plane) at the junction between the anterior third and posterior two-thirds of the spinal cord (in the sagittal plane); craniocaudal extension of less than three metamers; and no enlargement of the spinal cord at the level of the cavity (Figure 1). An atypical SC was any cavity not meeting the above three criteria (Figure 2).

Diffusion tensor imaging (DTI) metrics and spinal cross-sectional area were analyzed.

### Data Sources/Measurements and Quantitative Variables

Patients were clinically evaluated by a neurosurgeon and a physiotherapist.

MRI scans were acquired on an Achieva 3Tesla scanner (Philips, Amsterdam, Netherlands) with a multiple-element spine array coil that allowed parallel imaging. The MRI protocol was the same for all patients. Cavity size was determined by whole-spine high-resolution T2-weighted imaging (DRIVE). We also performed a 32-direction diffusion sequence with 3 mm-thick slices (voxel size: 2 × 2 × 3 mm) in the 1-h examination.

If the patient had a previous MRI performed at another center showing the SC, we considered this MRI as the baseline of the follow-up.

DTI analysis without spinal cord segmentation was performed on a voxel-by-voxel basis using DPTools software ^1^ as previously described (4).

#### DTI Analysis With Spinal Cord Segmentation

All diffusion-weighted images were corrected for motion artifacts using the sct_dmri_moco function in SpinalCordToolbox package^2^ (5). Gradient diffusion directions were also corrected according to the detected movements. To obtain a spinal cord mask, the average of non-diffusion images was thresholded using the Otsu method (6). Fractional anisotropy (FA) and mean diffusivity (MD) images were computed with the dtifit function of FMRIB Software Library package v.5.0 (7). Mean FA values per slice were computed in the spinal cord mask. A trained neurosurgeon matched each slice with the vertebral level and the mean FA per vertebra was computed for each level. The same steps were performed for MD maps. FA and MD values were compared to values obtained in 19 healthy volunteers (8). In axial sections of the upper cervical spine, mean FA was 0.45 (Standard Deviation [SD] = 0.018) and mean MD was 0.96 (SD = 0.083).

Spine cross-sectional area was measured from the T2-weighted images at the level of the C2 vertebra. Five 3 mm-thick slices perpendicular to the spinal cord were selected, with the most inferior slice passing centrally through the C2/C3 disk (9). The cord area was measured using a highly accurate semi-automated method (10) and compared with values in control subjects where the mean spinal cord diameter was 84.7 mm (9).

### Statistical Analysis

Statistical analysis was performed using R software^3^.

## Results

### Participants

Forty-eight consecutive patients were included in the study (29 females). The mean age at diagnosis was 41 years. Detailed information on the population is presented in Table 1.

**Table 1:** Comparison of our results with the literature.

	**Population**			**Clinical exam**					**Radiology**				**Evolution**			
**References**	**Number**	**Age**	**F/M**	**Axial pain**	**Radicular pain**	**Deficit**	**Trauma**	**Asymptomatic**	**Diameter (mm)**	**Span (vertebral levels)**	**location**	**Flow**	**Mean FU**	**Stable or improved**	**Worsening**	**enlargement**
Jinkins and Sener (2)	3	27 (18–35)	2/1	66%(2)	None	None	None	33% (1)	NA	2.6 (2–3)	Cervical: 33% (1) Thoracic: 66% (2)	NA	3 (2–4) years	All	No	No
Petit Lacour et al. (3)	12	34 (14–65)	10/2	58% (7)	58% (7)	8% (1)	16% (2)	0	2–4 (available in 2 patients, others = filiform)	4.6 (2–11)	Cervical: 16% (2) Thoracic: 58% (7) Both: 25% (3)	NA	9 years for one patient only	Yes (for one patient only)	No	No
Roser et al. (1)	40	36 (11–62)	25/15	#66%		0	NA	10%	2.7 (1.2–5.8)	3.5	Cervical: 23% Thoracic: 51% Both: 25%	normal	36.9 months (6–93)	All	No (worsening of pain)	No
Holly and Batzdorf (11)	32	40 (16–63)	14/18	41% (13)	22% (7)	44% (20) minor motor deficits	31% (10)	NA	2 (1–5)	3 (1–9)	16 cervical 12 thoracic 4 both (patients missing)	NA	38 months (6–110)	78% (25)	22% (7)	No
Magge et al. (12)	48	9.7 (0.2–19.3)	30/18	27% (13)		31% (15) (neurol. Symptoms)	NA	12% (6)	4 (1.2–9.4)	7.1 (2–17)	Cervical: 4% (2) Thoracic: 63% (30) Both: 33% (16)	NA	15.5 months (3–56)23.8 months (2–64)	*N* = 13 (in subgroup)	*N* = 2 (in subgroup)	NA *N* = 4 (in subgroup)
Klekamp (13)	635	40.8 ± 14.8	2/1	79.5%	NA	10%	NA	NA	NA	NA	Cervical: 23% Thoracic: 70% Both: 7%	normal	No follow-up			
Joseph et al. (14)	39	10.6 (3–16)	25/14	36% (14)	NA	NA	NA	64% (25)	3.30 (1.1–7)	2–19	Cervical 8% (3) Thoracic 84% (33) Both 8% (3)	NA	15.6 months (4–84)	97% (38)	3% (1)	No
Our study	48	44.7 (12–68)	29/19	60% (29)	48% (23)	15% (7)	NA	2% (1)	4.12 (1.5–7)	4.8 (1–18)	Cervical: 27% (13) Thoracic: 46% (22) Both: 27% (13)	NA	58 (6–344 m)	81% (39)	19% (9)	No

### Descriptive Data

All but one patient were symptomatic upon diagnosis, with one or more of the following symptoms at first consultation: axial pain (either cervical or lumbar) in 29 patients (60%), radicular pain in 23 patients (48%), and mild motor deficit (ASIA > 95/100) in seven patients (15%). No patient had urinary symptoms.

SCs were located in the spinal cervical region in 13 patients (27%), the spinal thoracic region in 22 patients (46%), and in both regions in 13 patients (27%). A typical SC was observed in 28 patients (58%) by MRI and in nine (19%), the cavity spanned more than three vertebrae. The spinal cord was slightly enlarged in 17 patients (35%). The cavity was located between the anterior third and posterior two-thirds of the spinal cord in all patients.

In 27 patients of this series there was a disc herniation at the level of the slitlike cavity (9 patients with atypical slits and 18 patients with typical slits) but only in 3 patients were the symptoms clearly related to degenerative disc disease, two of these patients had been operated on for cervical disc herniation. In one of these two patients the intramedullary slit was visible on preoperative imaging.

Among the 47 patients who presented with pain, correlation between symptoms and imaging was good for only 9 patients (19%). In 32 patients (68%) it was impossible to attribute all symptoms to the SC since there were symptoms above the cavity level and/or evidence of degenerative spine disease on imaging. In 6 patients (13%) the symptoms were clearly not related to the SC. This disparity was irrespective of the typical or atypical nature of the cavity: over 20 patients with atypical cavities, concordance was good for 5 patients, was intermediate for 12 patients, and was poor for 3 patients. For the 28 patients with typical cavities, concordance was good for 4 patients, was intermediate for 20 patients, and was poor for 4 patients.

Tractographic reconstructions were available for 34 patients. Visual inspection revealed abnormalities in 33 patients, with rarefaction and/or disorganization of fibers at the location of the cavity. Based on the assumption that the water content of the cavity could cause artifacts, we did not perform analyses at the level of the cavity; instead, we chose to analyze DTI metrics in the upper cervical cord including FA and MD, which were available for 32 and 31 patients, respectively. Mean FA was 0.38 (SD = 0.035; minimum[min] = 0.28; maximum [max] = 0.45); MD was 1.79 (SD = 0.25; min = 1.21; max = 2.37); mean transverse diffusivity (TD) was 1.32 (SD = 0.41; min = 0.56; max = 1.96); and mean axial diffusivity (AD) was 2.18 (SD = 0.72; min = 0.23; max = 2.83).

Given that these metrics could be influenced by perimedullary cerebrospinal fluid (CSF), we performed another analysis of the same data after automatic segmentation of the spinal cord which were available for 18 patients. Mean FA was 0.54 (SD = 0.07; min = 0.40; max = 0.64); MD was 1.41 (SD = 0.28; min = 1.04; max = 2.17); mean TD was 1.04 (SD = 0.34, min = 0.64, max = 1.86); and mean AD was 2.27 (SD = 0.31; min = 1.84; max = 3.17).

Spine cross-sectional area data were available for six patients. Mean cross-sectional area at the upper cervical level was 98 mm^2^ (SD = 16.72; min = 74.94; max = 115.66).

### Outcome Data

Mean follow-up from the time of diagnosis of the cavity was 58 months (SD = 64 months; min = 6 months; max = 344 months).

**Figure 3:**
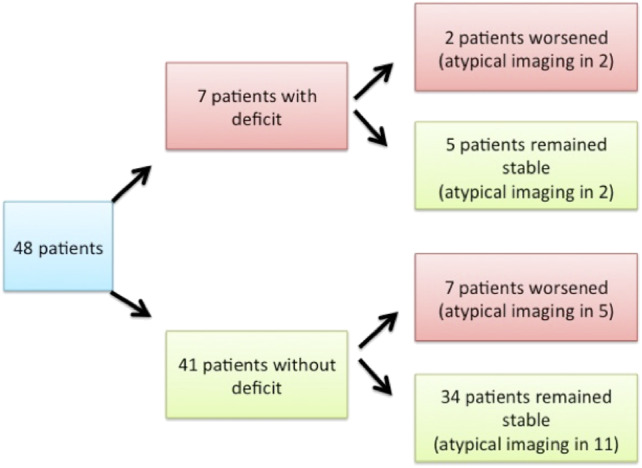
Clinical exam upon presentation and clinical evolution of patients.

At first consultation, seven patients presented with mild motor deficit (> 4/5); among them, two experienced worsening of the deficit or developed urinary symptoms, whereas five remained stable. Of the 41 patients who did not present any deficit at first consultation, seven developed mild motor deficit or urinary symptoms and 34 remained stable (Figure 3). The ASIA score for all patients was above 90/100. The level of pain remained stable in 31 patients (64%), worsened in seven (15%), and improved in three (6%). Data regarding the evolution of pain were not available for seven patients (15%).

MRI control scans with T2-DRIVE sequence were available for 42 patients; the cavity was stable in all patients.

## Main Results

### Relationship Between Clinical Data and MRI Features at Baseline

Among the 28 patients with typical SC and 20 with atypical SC, two (7,1%) and seven (35%) patients, respectively, experienced worsening of their condition, with the appearance of motor deficit or urinary symptoms. A statistically significant difference was observed between the two groups (*P* = 0.02; Fisher's exact test). The positive and negative predictive values were 0.35 and 0.93, respectively (Table 2).

**Table 2:** Relationship between clinical parameters and imaging.

***N* = 48**	**No worsening (*n* = 39)**	**Worsening (*n* = 9)**	
Typical imaging	26	2	*N* = 28
Atypical imaging	13	7	*N* = 20
	Positive predictive value = 0.35		*P* = 0.02
	Negative predictive value = 0.93		

Among the nine patients whose condition worsened (appearance of a new deficit or aggravation of an existing deficit), DTI metrics of the segmented cervical spinal cord were available for four. Mean FA was 0.575 (SD = 0.08; min = 0.46; max = 0.62) and MD was 1.410 (SD = 0.31; min = 1.04; max = 1.8). Among the 39 patients who remained stable (including those who presented with a deficit at first consultation), DTI metrics of the segmented cervical cord were available for 14; mean FA was 0.531 (SD = 0.07; min = 0.40; max = 0.64) and MD was 1.414 (SD = 0.28; min = 1.1; max = 2.17). Differences between the two groups were not statistically significant for FA (P =.3514, confidence interval: −0.069, 0.158; Student's *t*-test) or MD (*P* = 0.984, confidence interval: −0.454, 0.462; Student's *t*-test).

Among the nine patients whose condition worsened, data regarding the cross-sectional area of the spinal cord at the C2 level were available for three. The mean area was 100.26 mm^2^ (SD = 22.10; min = 74.94; max = 115.66). Among the 39 patients who remained stable, data were available for three; the mean area was 95.81 mm^2^ (SD = 13.99; min = 86.75; max = 111.92).

## Discussion

### Key Results

This is the largest study to prospectively assess clinical status and imaging findings in patients presenting with SCs. Our results revealed a different clinical course between patients with a typical and those with an atypical SC. Among the 28 patients in the former group, two worsened, with appearance of a mild motor deficit or urinary symptoms; among the 20 latter patients, seven experienced worsening of their condition. A typical intramedullary SC had very good negative predictive value (0.93), which can reassure patients. However, we did not identify any DTI imaging parameter that could predict an unfavorable evolution at the individual level.

### Interpretation

Our results are in accordance with the literature (1, 3, 11, 13) (Table 1). However, most studies did not report the development of a deficit, with one exception (11); and others have described experiences with pediatric patients (12, 14).

In our study, diffusion imaging was not predictive at the individual level. All patients presented with rarefaction of fibers at the location of the cavity, which was likely an artifact. Our results at the cervical level (decreased FA and increased MD) could be due either to axonal degeneration or to low signal-to-noise ratio (15). We also observed an increase in the cross-sectional area of the spinal cord in the most severe patients, although this is a very preliminary result based on a small sample size.

Diffusion imaging data for syringomyelia is scarce (Table 3). In a study of one patient with multiple sclerosis and a cervical cavity, fiber tracks surrounding the cavity were described as normal and no DTI metrics were reported (16). An analysis of FA in syringomyelia patients revealed decreased FA relative to controls at the location of the cavity but normal FA value above and below the cavity (17), which is in contrast with our results and another study in patients with syringomyelia where authors found decreased FA at the cervical level compared to controls (8); this pattern appeared to predominate in the anterior cord, and the authors suggested that it was linked to changes in the spinothalamic pathways. Diffusion imaging of the spinal cord has technical limitations such as artifacts related to bone and respiratory and circulatory movements (18). The field is now moving toward higher-resolution imaging that allows atlas-based segmentation of fibers tracts in the spinal cord (19), which would enable the determination of correlations between neurological signs/symptoms and imaging findings (18).

**Table 3:** Comparison of our preliminary Diffusion Tensor Imaging data with the literature.

**References**	**Pathology**	**Fibertracking**	**Cervical level (patients/control)**				**Above cavity (patients/control)**				**Cavity (patients/control)**				**Below cavity (patients/control)**			
			**FA**	**MD**	**TD**	**AD**	**FA**	**MD**	**TD**	**AD**	**FA**	**MD**	**TD**	**AD**	**FA**	**MD**	**TD**	**AD**
Agosta et al. (16)	Syrinx (MS)	Normal	NA	NA	NA	NA	NA	NA	NA	NA	NA	NA	NA	NA	NA	NA	NA	NA
Roser et al. (17)	SC/control	NA	NA	NA	NA	NA	0,53/0,52	NA	NA	NA	0,19	NA	NA	NA	NA	NA	NA	NA
Hatem et al. (8)	Syringomyelia/control	NA	0,39/0,45	0,90/0,96	NA	NA	NA	NA	NA	NA	NA	NA	NA	NA	NA	NA	NA	NA
Our study	SC	Artifact in all patients	0,38	1,79	1,32	2,18	NA	NA	NA	NA	NA	NA	NA	NA	NA	NA	NA	NA
Our study after segmentation		NA	0,54	1,41	1,04	2,27	NA	NA	NA	NA	NA	NA	NA	NA	NA	NA	NA	NA

### Limitations

There were limitations to our study. The length of the follow-up was different for each patient. Importantly, we did not precisely measure chronic pain using appropriate scales. In terms of imaging, DTI sequences were not performed for all patients, and we did not obtain follow-up data for DTI imaging parameters for any patient. It would be interesting to investigate whether the evolution of DTI parameters mirrors clinical course (i.e., worsening of DTI parameters as the patient's condition deteriorates). Moreover, the DTI imaging was not of sufficiently high resolution to permit analysis of specific tracts, thereby precluding correlational analyses between imaging and neurological findings.

## Conclusion

Based on clinical and imaging parameters, it remains difficult to distinguish at the individual level between patients who will remain stable and those who will experience neurological worsening. However, we showed that most patients with a typical SC remain stable. Accordingly, these patients should be reassured at first consultation and managed by yearly clinical and MRI follow-up. On the other hand, symptomatic patients or those showing atypical SC should be managed by a multimodal approach (pain specialists, neurologists, and rheumatologists), with closer follow-up. We believe that technical improvements in imaging and electrophysiology will help identify the few SC patients with poor neurological outcome.

## Data Availability Statement

The datasets generated for this study will not be made publicly available. The dataset will be made available for any reasonable request, every request for dataset should be sent to corresponding author.

## Ethics Statement

The studies involving human participants were reviewed and approved by the study was approved by the Ethics Committee of the Pitié-Salpêtrière Hospital (ID RCB: 2013-A01547-38, Paris, France). Written informed consent to participate in this study was provided by the participants' legal guardian/next of kin.

## Author Contributions

MF and NA substantially contributed to the conception and design of the study, to the acquisition of data, and to the analysis and interpretation of data, and were involved in drafting the manuscript or revising it critically for important intellectual content and in producing the approved final version for publication. DD performed acquisition of MRI data. MF, DD, ME-M, and SD performed analysis of imaging data and wrote methodological section regarding each imaging modality. FP, NA, and SM reviewed the final manuscript. All agreed to be accountable for all aspects of the work by ensuring that questions related to the accuracy or integrity of any part of the work are appropriately investigated and resolved.

## Conflict of Interest

The authors declare that the research was conducted in the absence of any commercial or financial relationships that could be construed as a potential conflict of interest.

## Footnotes

^1^http://fmritools.com

^2^https://sourceforge.net/p/spinalcordtoolbox/wiki/sct_dmri_moco/)

^3^R Core Team. R: A language and environment for statistical computing. Vienna, Austria. *R Foundation for Statistical Computing*. 2017. http://www.R-project.org/)
